# Enological potential of non-*Saccharomyces* yeast strains of enological and brewery origin from Ukrainian Collection of Microorganisms

**DOI:** 10.1080/21501203.2020.1837272

**Published:** 2020-11-29

**Authors:** Olga Ianieva, Valentin Podgorsky

**Affiliations:** Department of Physiology of Industrial Microorganisms, Zabolotny Institute of Microbiology and Virology, Kyiv, Ukraine

**Keywords:** Non-*Saccharomyces* yeasts, enological traits, hydrolytic activities, stress tolerance, fermentative activity

## Abstract

Non-conventional wine yeasts are extensively studied as promising producers of hydrolytic enzymes and as potential starter cultures in winemaking due to their ability to improve organoleptic properties of wine. Thirty-six yeast strains of enological and brewery origin from the Ukrainian Collection of Microorganisms belonging to *Torulaspora, Kloeckera, Candida, Metschnikowia, Pichia*, and *Zygosaccharomyces* genera have been screened for the production of extracellular hydrolases, stress tolerance, fermentative activity, and other traits of enological interest. This study revealed the high incidence of lipolytic, proteolytic, and β-glucosidase activities among the yeasts, while no pectinase activity was detected. Esterase, cellulase and glucanase activities were found in a small proportion of yeasts (8.33–16.66%). Several *Pichia anomala, Kloekera javanica, Pichia membranifaciens*, and *Metschnikowia pulcherrima* strains demonstrated a wide range of hydrolytic activities. High tolerance to stress factors (ethanol, osmotic, and oxidative stress) present during alcoholic fermentation was detected in *P. anomala* and *M. pulcherrima* strains. Fermentative activity of several yeast strains was evaluated in microfermentations in a model semi-synthetic medium. Strain *P. anomala* UCM Y-216 was selected as the most promising culture for winemaking due to its hydrolytic activities, tolerance to stress factors and other valuable metabolic traits. This study represents the first step for selecting a non-conventional yeast strain of enological origin as a potential co-culture for winemaking.

## Introduction

Most studies researching biodiversity and biotechnological application of yeasts in winemaking and brewing focus on *Saccharomyces cerevisiae* – yeasts traditionally used in these industries. It is a well-known fact that *Saccharomyces* yeasts are found considerably rarer and in lesser numbers on grapes surface compared to other yeast species (Barata et al. 2012). If wine is produced by spontaneous fermentation there is a greater diversity of aerobic and weakly fermenting yeasts that are predominant in grapes juice or must that include *Cryptococcus, Debaryomyces, Issatchenkia, Kluyveromyces, Pichia, Rhodotorula*, and *Zygosaccharomyces* (Varela and Borneman 2017). However, most of these yeasts are gradually replaced by more ethanol-tolerant yeasts with high fermenting activity. Some non-*Saccharomyces* yeasts can still survive at high ethanol concentrations and take part in fermentation, however, the major part of ethanolic fermentation at later stages is conducted by *Saccharomyces* yeasts (Fleet 2003). Nowadays, specially selected *S. cerevisiae* strains are used in alcoholic fermentation to reduce the participation of the wild yeast microbiota that can produce undesirable compounds such as biogenic amines or excessive amounts of organic acids and often are the cause of wine spoilage (Padilla et al. 2016). However, non-*Saccharomyces* yeasts can also produce a number of secondary metabolites that can considerably improve the organoleptic characteristics of the final product (Ivit and Kemp 2018).

Grapes as a substrate for alcoholic fermentation contain a broad spectrum of various chemical compounds including pectins, cellulose, hemicelluloses, glucans, proteins, lignin, phenolic substances, various aromatic precursors (Claus and Mojsov 2018). The degradation of these components could lead to the improvement of wine clarity, taste and aroma. The ability to degrade a wide range of organic polymers present in grapes would be an important and desired characteristic for the potential wine yeast culture (Jolly et al. 2014). Many non-conventional yeasts possess hydrolytic enzymes that are lacking in *Saccharomyces* yeasts that allow them to improve the taste and aroma, and, as a result, the complexity of wine (Escribano et al. 2017).

Recently non-conventional yeasts attracted considerable attention due to their killer and enzymatic acivities and their role in the formation of aroma and flavor in wine . A number of non-*Saccharomyces* yeasts were investigated as potential co-starter cultures in winemaking including *Metschnikowia pulcherrima* (Barbosa et al. 2018), *Saccharomycodes ludwigii* (Esteves et al. 2019), *Torulaspora delbrueckii* (Tataridis et al. 2013), *Hanseniaspora osmophila* (Viana et al. 2008), *Wickerhamomyces anomalus* (Izquierdo Cañas et al. 2014) and some others. Several yeast strains of enological origin were proposed as potential starter cultures to obtain wines with reduced ethanol content: *Hanseniaspora uvarum, H. osmophila, Starmerella bacillaris* and *Candida membranaefaciens* (Mestre Furlani et al. 2017), *M. pulcherrima, Schizosaccharomyces malidevorans, Candida stellata* (Contreras et al. 2014), *M. pulcherrima* and *Saccharomyces uvarum* (Varela and Borneman 2017). Also non-conventional yeasts of enological origin could be exploited to lower wine acidity (Vilela 2019). Consequently, there is notable interest in the search and selection of promising non-conventional wine yeasts, and investigating their enological characteristics, i.e. enzymatic and metabolic characteristics, tolerance to various stress factors that could be present during fermentation process, fermenting activity, safety, killer activity, etc. (Jolly et al. 2014).

The Ukrainian Collection of Microorganisms contains almost 1500 yeast strains isolated from various sources which include grapes, grape juice, must, wine, beer and, wort. Non-conventional yeasts isolated from these habitats could possess valuable enological characteristics that could be exploited for the production of wines, especially with reduced ethanol content as most non-*Saccharomyces* yeasts possess lower fermentative power compared to *S. cerevisiae* (Ciani et al. 2016).

The aim of this study was to evaluate non-conventional strains of enological and brewery origin from the Ukrainian Collection of Microorganisms for traits of enological interest as potential starter cultures or co-cultures in winemaking.

## Materials and methods

### Yeast strains and inoculum preparation

36 yeast strains of enological and brewery origin used in this study are listed in Table 1. They were obtained from the Ukrainian Collection of Microorganisms, Institute of Microbiology and Virology, Kyiv, Ukraine. The yeast strains were maintained by subculturing every 8–12 months on malt agar medium (Kurtzman et al. 2011) and stored at 4–6°C.

**Table 1.** Yeast strains used in the study

Yeast species	Strain number UCM Y-		Strain origin	Yeast species	Strain number UCM Y-	Strain origin
*Torulaspora delbrueckii*	2737	wine, Kyiv		*Pichia membranifaciens*	471	wine, Chisinau, Moldova
*T. delbrueckii*	2738	wine, Kyiv		*P. membranifaciens*	473	wine, Chisinau, Moldova
*T. delbrueckii*	2739	wine, Kyiv		*P. membranifaciens*	477	wine, Chisinau, Moldova
*T. delbrueckii*	2741	wine, Kyiv		*P. membranifaciens*	480	wine, Chisinau, Moldova
*T. delbrueckii*	2749	wine, Odesa		*P. membranifaciens*	481	wine, Chisinau, Moldova
*Kloeckera apiculata*	2728	brewery, Kyiv		*P. membranifaciens*	482	wine, Chisinau, Moldova
*K. apiculata*	2729	brewery, Kyiv		*P. membranifaciens*	2733	wine, Kyiv
*K. javanica*	2689	wine, Odesa		*P. membranifaciens*	2734	wine, Kyiv
*K. javanica*	2693	brewery, Kyiv		*P. membranifaciens*	2735	wine, Kyiv
*K. javanica*	2695	brewery, Kyiv		*P. anomala*	212	wine, Chisinau, Moldova
*Candida inconspicua*	2732	wine, Kyiv		*P. anomala*	213	wine, Chisinau, Moldova
*C. lambica*	2696	brewery, Kyiv		*P. anomala*	215	wine, Chisinau, Moldova
*C. valida*	969	wine, Chisinau, Moldova		*P. anomala*	216	wine, Chisinau, Moldova
*C. valida*	971	wine, Chisinau, Moldova		*P. anomala*	217	wine, Chisinau, Moldova
*C. valida*	973	wine, Chisinau, Moldova		*Zygosaccharomyces bailii*	657	grapes juice, Chisinau, Moldova
*C. vini*	997	wine, Chisinau, Moldova		*Z. bisporus*	2730	spoilt wine
*C. vini*	998	wine, Chisinau, Moldova		*Z. bisporus*	2731	spoilt wine
*Metschnikowia pulcherrima*	333	grapes, Chisinau, Moldova		*Z. fermentati*	658	grapes juice, Chisinau, Moldova

For killer activity yeast strains *S. cerevisiae* UCM Y-554 and *Kluyveromyces marxianus* UCM Y-1591 (CBS Y-712) were used as killer-sensitive strains and yeast strains *S. cerevisiae* UCM Y-2505 and UCM Y-522 were used as positive controls for killer activity. Yeast strains were obtained from the Ukrainian Collection of Microorganisms.

Yeast strains were cultivated on YPD agar containing 1% yeast extract, 2% glucose, 2% peptone, and 2% agar (w/v) for 2–3 days at 25–26°C and yeast suspensions were made in sterile 0.9% NaCl solution to a final cell concentration of approximately 10^6^ CFU/ml and were replica plated on the appropriate media using a multi-point steel inoculator.

### Qualitative determination of extracellular hydrolytic activities

Proteolytic activity was assessed using gelatin as a substrate. Gelatin hydrolysis was assessed in Yeast Nitrogen Base (YNB) broth containing 0.5% glucose and 10% gelatin for 3 weeks at 25–26°C (Kurtzman et al. 2011). The proteolytic activity was examined every week by placing gelatin tubes into the fridge for 1 h until the solidification of the medium and checking afterwards for the signs of gelatin liquefaction. The liquefaction of gelatin indicated the presence of proteolytic activity.

Cellulolytic activity was determined on YPD agar supplemented with 0.5% carboxymethylcellulose (CMC), pH 6.0. Plates were incubated at 25–26°C for 7 days and stained with 0.03% Congo Red followed by destaining with 1 M NaCl. The formation of the hydrolysis zone around colonies indicated the presence of cellulolytic activity (Strauss et al. 2001).

Lipolytic activity was determined on tributyrin agar (0.5% peptone, 0.3% yeast extract, 1% tributyrin, 1.5% agar, final pH 6.0). Plates were incubated for 7 days at 25–26°C (Brizzio et al. 2007). The appearance of the clear zone of more than 1 mm from the edge of the yeast colony indicated positive lipolytic activity, the appearance of the clearing less than 1 mm from the colony edge or under the colony was considered as a weak activity, while the absence of any changes indicated a negative result (Charoenchai et al. 1997).

Esterase activity (the ability to hydrolyse long-chain esters) was assessed by plating yeasts on tween-80 agar (1% peptone, 0.5% NaCl, 0.01% CaCl_2_х2H_2_O, 1% tween-80, 2% agar). Plates were incubated at 25–26°C for 1 week. The formation of precipitate around yeast colonies more than 1 mm in width demonstrated positive esterase reaction, the appearance of the precipitation zone less than 1 mm from the edge of the colony was considered as weak activity, the absence of the zone indicated the lack of esterase activity (Charoenchai et al. 1997).

Xylanase activity was determined according to Strauss et al. (2001) with some modifications on YNB agar containing 1% xylan. Plates were incubated at 25–26°C for 7 days and flooded with iodine solution. The formation of a hydrolysis zone (clearing) around colonies indicated the presence of xylanase activity.

Pectinase activity was determined on YNB agar supplemented with 1% citrus pectin (Brizzio et al. 2007). Plates were incubated at 25–26°C for 7 days and flooded with iodine solution (Martinez et al. 2016). A clear halo (hydrolysis zone) around yeast colonies indicated the presence of pectinase activity.

β-glucosidase activity was determined on an agar medium containing 0.5% arbutin, 1% yeast extract, 2% agar. After sterilisation, 2 ml of 1% ammonium ferric citrate solution was added to the 100 ml medium (Kurtzman et al. 2011). 100 µl yeast suspension, prepared as indicated earlier, was added to the tubes containing the agar medium. Tubes were incubated at 25–26°C for 5–7 days. The change in the colour of the medium to dark purple-brownish was considered as a positive reaction, while the change to light-medium brown was considered as weak activity. The test for β-glucosidase activity was conducted in 10 ml tubes, as incubation of yeasts on agar plates led to false positive results.

### Tolerance to stress factors

Yeast suspensions were replica plated on agar medium containing the corresponding stress factor (glucose, ethanol, copper sulphate). YPD medium without stress factor was used as a positive control.

Osmotolerance of yeasts was determined on 50% glucose YPD agar (Kurtzman et al. 2011). Plates were incubated for 2 weeks at 25–26°C. Ethanol tolerance was determined according to Barbosa et al. (2018), with some modifications: on YPD agar containing ethanol at 6%, 9%, 12% and 16% concentrations at pH 6.0 and 3.5, respectively, to imitate the conditions of alcoholic fermentation. Plates were incubated for 1 week at 25–26°C. Yeast tolerance to copper was evaluated on YPD agar containing 200 and 400 µM CuSO_4_, pH 6.0 and 3.5 (Capece et al. 2018). Actively growing strains under such conditions were considered tolerant, while poor yeast growth was regarded as weak tolerance and the absence of the growth indicated the lack of tolerance to the corresponding stress factor.

Oxidative stress tolerance of yeast strains was evaluated by testing yeast tolerance to hydrogen peroxide according to Mestre Furlani et al. (2017), with some modifications. 0.1 ml of yeast suspensions was spread on the surface of YPD agar plate. Wells (7 mm in diameter) were made using a sterilised borer and 50 µl of H_2_O_2_ at concentrations of 250 μM, 500 μM and 1 mM, respectively, were added into each well. Plates were incubated for 48 h at 25–26°C and the average diameter (mm) of the inhibition zone around the wells was measured.

### Assessment of enological traits of yeasts

Acid production from glucose by yeast strains was evaluated on Causter’s chalk agar containing 5% glucose, 0.5% yeast extract, 0.5% CaCO_3_, 2% agar (Kurtzman et al. 2011). Plates were incubated for 1 week at 25–26°C. The appearance of the clearing zone as a result of chalk solubilisation indicated the ability of yeasts to produce organic acids.

Production of biogenic amines by yeast strains was tested on YPD agar medium containing a mix of amino acids with a total concentration of 1% or 2% and 0.0015% bromocresol purple, final pH 5.2 (Aslankoohi et al. 2016). The following amino acids were added to the medium at equal ratios: tyrosine, histidine, phenylalanine, leucine, tryptophan, arginine, and lysine. Plates were incubated for 7 days at 25–26 °C. The medium lacking amino acids and inoculated with yeasts was used as a negative control. Biogenic amine production was indicated by the appearance of purple halo around the colonies. Yeasts were qualitatively defined as weak or positive biogenic amine producers according to the intensity of the produced colour.

Malic and acetic acid assimilation by yeast strains was tested according to Šuranská et al. (2016) on YNB agar (0.67%) containing 0.5% malic acid and 0.25% (w/v) acetic acid. Yeasts were cultivated for up to 3 weeks at 25–26°C.

The killer phenotype of the yeast strains was assessed according to Raymond Eder et al. (2017), with some modifications. Killer-sensitive yeast strains *S. cerevisiae* UCM Y-554 and *K. marxianus* UCM Y-1591 were inoculated into YPD-methylene blue agar containing 0.003% (w/v) methylene blue, pH 4.5 at the final cell concentration of approximately 10^5^ CFU/ml. Yeast strains were streaked on the agar seeded with the sensitive test strain and incubated at 25–26°C for 48–72 h. Killer activity was considered positive if a zone of growth inhibition or a region of methylene blue-stained dead yeast cells around the streaks of the tested yeast strains were observed.

### Fermentative activity

The fermentative potential of yeast strains was preliminarily tested in bent fermentation tubes filled with semi-synthetic fermentation medium containing 20% sucrose, 1% yeast extract, 0.1% (NH_4_)_2_SO_4,_ 0.1% KH_2_PO_4,_ 0.1% MgSO_4_ (Lopes et al. 2007). The pH of the medium was adjusted to 3.5, similar to wine, using tartaric acid. Tubes were incubated at 25–26°C for 7–14 days, and fermentation activity of yeast strains was qualitatively assessed by the amount of carbon dioxide gas accumulated in the closed arm of the fermentation tube.

Microfermentations were conducted in 150 ml Erlenmeyer flasks containing 100 ml of the same semi-synthetic fermentation medium adjusted to pH 3.5. Yeast inoculum for microfermentations was obtained from 48 h yeast cultures grown in YPD broth. Flasks were inoculated with the selected yeast strains to give the final cell density of approximately 10^6^ CFU/ml and stoppered with glass fermentation traps containing 40% sulphuric acid to allow only CO_2_ to escape the fermentation medium. Fermentation was conducted under static conditions (without shaking) at 25°C. The weight loss of the flasks due to CO_2_ production was measured daily. Microfermentations for each strain were conducted in triplicate.

### Statistics

The qualitative tests were done in triplicate and the results were assessed as positive, weak, or negative if at least 2 out of 3 replicates produced the same result. The results of the quantitative experiments are presented as means of triplicates with the corresponding standard deviation (± SD). The means were compared by the one-way analysis of variance (ANOVA), p < 0.05 was regarded as statistically significant.

## Results and discussion

In this work, 36 yeast strains isolated from sites of enological and brewery origin and maintained in the Ukrainian Collection of Microorganisms were screened for valuable biotechnological traits for winemaking, including the production of extracellular hydrolases, stress tolerance, production of organic acids, biogenic amines, killer activity and fermentative activity.

**Figure 1.**
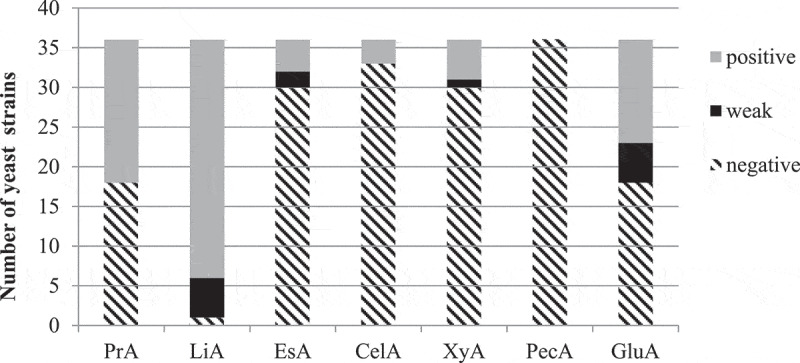
Extracellular hydrolytic activities of yeast strains of enological and brewery origin: PrA – proteolytic activity (gelatin hydrolysis), LiA – lipolytic activity (tributyrin hydrolysis), EsA – esterase activity (tween-80 hydrolysis), CelA – cellulolytic activity (CMC hydrolysis), PecA – pectinase activity, GluA – β-glucosidase activity

The results of the screening of 36 yeast strains for the production of extracellular hydrolytic enzymes are summarised in Figure 1. One of the most relevant enzymes of the enological value is β-glucosidase that breaks down glycosidic complexes, thus releasing terpenes and other volatile compounds (Claus and Mojsov 2018). β-glucosidase activity was demonstrated by the hydrolysis of arbutin that resulted in the appearance of light or dark brown-purple colour in the medium. As neighbouring colonies with strong β-glucosidase activity very often caused the darkening of the surrounding medium and to false positive results, tests were conducted in 10 ml tubes. Thirteen out of 36 yeast strains belonging to *Zygosaccharomyces fermentati, Z. bailii, Pichia anomala, P. membranifaciens, M. pulcherrima, C. inconspicua, K. javanica*, and *K. apiculata* possessed strong β-glucosidase activity. Eighteen out of 36 yeast strains did not exhibit β-glucosidase activity, 5 strains demonstrated weak activity.

A high incidence of β-glucosidase activity among yeasts of enological origin was observed in several works, especially among *Hanseniaspora* spp, *Meyerozyma (Pichia) guilliermondii, Wickerhamomyces (Pichia) anomalus, Metchnikowia* spp. and *Rhodosporidium toruloides* (Belda et al. 2016), *T. delbrueckii, Lachancea thermotolerans*, and *M. pulcherrima* (Comitini et al. 2011), *Hanseniaspora guilliermondii, T. delbrueckii, M. pulcherrima*, and *Saccharomycodes ludwigii* (Grazia et al. 2019). Among 97 non-conventional wine yeasts, the highest proportion of strains with β-glucosidase activity was detected in *M. pulcherrima* (63%) and *Cryptococcus* spp. (60%) (Escribano et al. 2017). However, interestingly none of 245 wine yeasts possessed the ability to hydrolyse arbutin as reported by Strauss et al. (2001). Fernández et al. (2000) detected β-glucosidase activity only in 14% wine yeasts, predominantly in *M. pulcherrima* strains. So we can conclude that, while some non-*Saccharomyces* yeast species like *M. pulcherrima* tend to possess β-glucosidase activity, this characteristic is mostly strain-dependent.

The most commonly detected extracellular enzyme in this study was lipase. Lipolytic activity was detected in 35 out of 36 yeast strains, only one *T. delbrueckii* strain lacked the ability to hydrolyse tributyrin. The most strongly lipolytic strains belonged to *P. anomala*, producing hydrolysis zones that were 38–50 mm in diameter (data not shown). A much smaller proportion of the yeasts (16.67%) exhibited esterase activity, i.e. the ability to hydrolyse short-chain esters using tween-80 as a substrate, strains belonging to *P. anomala, P. membranifaciens, C. vini*, and *K. javanica*. These results are in agreement with other studies, which reported a high incidence of lypolytic activity and the comparable frequency of esterase activity in ascomyceteous yeasts isolated from tropical environments (Buzzini and Martini 2002) and *Pichia/Wickerhamomyces* yeasts isolated from enological ecosystems (Madrigal et al. 2013). However, in another study the ability to hydrolyse tributyrin was observed only in 27.27% non-conventional yeasts belonging to *T. delbrueckii, C. pulcherrima, C. stellata* and *C. krusei* (Charoenchai et al. 1997). Although the possession of lipolytic activity is not essential for the promising wine culturew, lipolytic enzymes could degrade lipids that originated from grapes or were the result of autolysis of yeasts thus releasing free fatty acids into wine and improving the quality of wine (Claus and Mojsov 2018).

Half of the yeast strains demonstrated the ability to hydrolyse gelatin, i.e. proteolytic activity, while none of *P. anomala, Z. bailii*, and *Z. bisporus* strains were proteolytic. The most strongly proteolytic strains were *M. pulcherrima* Y-333, *T. delbrueckii* Y-2737 and Y-2741, *P. membranifaciens* Y-473, and completely hydrolysed gelatin by the fourth day of cultivation. In a large study conducted with 770 wine yeast strains protease activity together with β-glucosidase activity was the most frequently observed enzymatic activity, and mostly in *Metschnikowia* and *Hanseniaspora* strains (Belda et al. 2016). Similar findings were reported by Binati et al. (2019) for 104 yeast strains from high-sugar habitats belonging to *Starmerella, Lachancea* and *Metschnikowia*, where 90% of *Metschnikowia* isolates possessed proteolytic activity. However, a low incidence of proteolytic activity was observed by Fernández et al. (2000) in 182 non-conventional wine yeasts, mostly in *P. membranifaciens* and *M. pulcherrima*, while none of 34 wine yeasts of the genera *Candida, Lachancea (Kluyveromyces), Metschnikowia*, and *Torulaspora* exhibited proteolytic activity on milk agar (Comitini et al. 2011). Such discrepancies could be partly explained by the use of different substrates and media in different studies. For example, in the study performed by Mautone et al. (2010) on 446 yeast and yeast-like strains isolated from phylloplane, 170 yeast strains hydrolysed casein, while only 72 yeasts possessed gelatinase. Proteolytic enzymes of non-*Saccharomyces* yeasts can play an important role in reducing haze caused by proteins in wine and beer, as *S. cerevisiae* usually does not possess extracellular proteolytic activity (Claus and Mojsov 2018).

Cellulose and hemicellulose are the key structural components of the cell wall of plants so their hydrolysis would result in the release of various aromatic and pigmented compounds from the grape skin and improvement of aroma and colour of wine (Claus and Mojsov 2018). Cellulolytic and xylanolytic activities are rarely observed in yeasts and in this study only a small proportion of strains exhibited the ability to degrade carboxymethylcellulose (8.33%), i.e. *K. apiculata, K. javanica* and *P. membranifaciens*, and xylan (16.66%) – *P. anomala* and *M. pulcherrima* strains, thus confirming this fact. Similarly, Belda et al. (2016) detected cellulase activity only in *Aureobasidium pullulans* strains, and Strauss et al. (2001) detected xylanase activity only in 6 out of 245 yeasts of enological origin (*C. stellata, C. oleophila, C. pulcherrima, C. pelliculosa* and *K. apiculata*), while cellulase activity was found in 11 isolates (*C. stellata, C. pulcherrima* and *K. apiculata*). Among 17 *Pichia/Wickerhamomyces* enological isolates, 8 strains possessed xylanase activity (Madrigal et al. 2013). A large screening for extracellular hydrolases among yeasts isolated from the malting ecosystem revealed that among ascomycetous yeasts xylanase activity was found only in *A. pullulans* strains, while cellulase was detected in *A. pullulans, Geotrichum silvicola* and *Exophiala dermatidis*. Overall the ability to degrade complex polysaccharides was mostly restricted to basidiomycetes (Laitila et al. 2006). As the current study employed only ascomycetous yeasts the low incidence of cellulase and xylanase activity is not surprising.

Pectinolytic enzymes can play role in the degradation of polysaccharides of the plant cell wall of the grape skin and pulp improving clarification of wine and releasing aromatic and pigmented compounds (Claus and Mojsov 2018). None of the studied yeast strains possessed pectinolytic activity. Pectinolytic activity is rarely found in yeasts, especially in ascomycetes as used in this study. Out of 48 yeasts isolated from grapes only 11 belonging to *A. pullulans* and basidiomycetous yeasts were pectinolytic (Merín et al. 2015). None of the 22 wine yeasts belonging to *Candida, Debaryomyces, Hanseniaspora, Hansenula, Kloeckera, Metschnikowia, Pichia, Saccharomyces* and *Torulaspora* exhibited pectinase activity (Charoenchai et al. 1997).

All the yeast strains possessed at least one extracellular activity of enological interest, while the majority of yeasts exhibited two or more hydrolytic activities (Table 2). The most frequent combination of enzymatic activities was lipolytic + proteolytic activities and was found in 10 yeast strains (27.7%) of *P. membranifaciens, C. valida, C. lambica*, and *T. delbrueckii*. 15 yeast strains possessed 3 or 4 hydrolytic activities, 3 out of 5 *P. anomala* strains exhibiting lipase, esterase, xylanase, and β-glucosidase activity. Similar findings were reported by Fernández et al. (2000) and Belda et al. (2016) in large screening studies of non-conventional yeasts of enological origin, although the prevalence of other enzymatic activities is reported. A wide range of extracellular hydrolases was demonstrated by *Pichia/Wickerhamomyces* enological isolates, including β-glucosidase, protease, esterase, pectinase and xylanase (Madrigal et al. 2013).

**Table 2.** Distribution of enzymatic activities among different species of yeasts of enological and brewery origin

Yeast species	Number of strains	Enzymatic activity*												
		PrA	LiA	LiA+PrA	LiA+ GluA	LiA+ PrA +GluA	LiA+ EsA+ GluA	LiA +PrA +CelA	LiA +GluA +CelA	LiA+ XyA +GluA	LiA+ EsA+ GluA+ CelA	LiA+ PrA +GluA+ XyA	LiA +EsA +GluA+ XyA	LiA+ EsA+PrA+GluA
*Torulaspora* *delbrueckii*	5	1	1	2	1	-	-	-	-	-	-	-	-	-
*Kloeckera apiculata*	2	-	-	-	1	-	-	-	1	-	-	-	-	-
*K. javanica*	3	-	-	-	1	1	-	-	-	-	1	-	-	-
*Candida inconspicua*	1	-	-	-	-	1	-	-	-	-	-	-	-	-
*C. lambica*	1	-	-	1	-	-	-	-	-	-	-	-	-	-
*C. valida*	3	-	2	1	-	-	-	-	-	-	-	-	-	-
*C. vini*	2	-	1	-	-	-	1	-	-	-	-	-	-	-
*Metschnikowia pulcherrima*	1	-	-	-	-	-	-	-	-	-	-	1	-	-
*Pichia membranifaciens*	9	-	-	6	-	1	-	1	-	-	-	-	-	1
*P. anomala*	5	-	-	-	-	-	-	-	-	2	-	-	3	-
*Zygosaccharomyces bailii*	1	-	-	-	1	-	-	-	-	-	-	-	-	-
*Z. bisporus*	2	-	2	-	-	-	-	-	-	-	-	-	-	-
*Z. fermentati*	1	-	-	-	-	1	-	-	-	-	-	-	-	-
Total	36	1	6	10	4	4	1	1	1	2	1	1	3	1

* PrA – proteolytic activity (gelatin hydrolysis), LiA – lipolytic activity (tributyrin hydrolysis), GluA – β-glucosidase activity (arbutin hydrolysis), EsA – esterase activity (tween-80 hydrolysis), CelA – cellulase activity (CMC hydrolysis), XyA – xylanase activity (xylan hydrolysis)

Yeast strains were assessed for the ability to withstand several stress factors. The ability to tolerate ethanol at 6–16% (v/v) at pH 6.0 and 3.5 (to imitate conditions of alcoholic fermentation) was studied on YPD agar plates. The ability of yeast strains to grow at pH 3.5 was tested and, all the yeasts grew well at low pH (data not shown). The current study showed some variability in ethanol tolerance in different non-conventional yeast species. All the yeast strains could tolerate ethanol at 6% at pH 6.0 and all but one at pH 3.5 (Table 3). 25 out of 36 strains in this study managed to survive at ethanol concentrations typically found by the end of alcoholic fermentation (12–16%) at pH 6.0; however, at pH 3.5 this number decreased to 22 strains, mostly *Pichia* and *T. delbrueckii* strains. *K. apiculata, K. javanica*, and *C. vini* strains were the least tolerant to ethanol, while the most tolerant strain was *Z. bisporus* Y-2730 that tolerated 16% ethanol at pH 6.0 and 3.5.

**Table 3.** Ethanol tolerance of non-conventional yeast strains

Yeast species	Number of strains	Ethanol concentration (%, v/v)							
		pH 6.0				pH 3.5			
		6%	9%	12%	16%	6%	9%	12%	16%
*T. delbrueckii*	5	-	1	4	-	-	1	4	-
*K. apiculata*	2	1	1	-	-	2	-	-	-
*K. javanica*	3	3	-	-	-	2	-	-	-
*C. inconspicua*	1	-	-	-	1	-	-	1	-
*C. lambica*	1	-	-	1	-	-	-	1	-
*C. valida*	3	-	1	2	-	-	3	-	-
*C. vini*	2	2	-	-	-	1	-	-	-
*M. pulcherrima*	1	-	-	1	-	1	-	-	-
*P. membranifaciens*	9	-	-	9	-	-	-	9	-
*P. anomala*	5	-	-	4	1	-	-	5	-
*Z. bailii*	1	-	1	-	-	-	1	-	-
*Z. bisporus*	2	-	-	1	1	-	-	1	1
*Z. fermentati*	1	-	1	-	-	-	1	-	-
Total	36	6	5	22	3	6	6	21	1

Many non-conventional wine yeasts lack the ability to withstand high ethanol levels that allows *S. cerevisiae* strains to survive and dominate during grape fermentation (Fleet 2003). A large study of phenotypic diversity among non-*Saccharomyces* yeasts demonstrated high ethanol tolerance of *T. delbrueckii, W. anomalus* and *Z. bailii* species (Mukherjee et al. 2017), which is in agreement with the results reported in this study. *W. anomalus* is well known as a ubiquitous yeast species able to survive under a wide range of extreme environmental conditions including pH, temperature and osmotic stress (Mukherjee et al. 2017).

Important criteria for potential starter cultures for winemaking also include tolerance to osmotic stress, oxidative stress and copper sulfate that is often used to fight against fungal diseases of vine. Tolerance to oxidative stress was tested by the addition of different concentrations of hydrogen peroxide and was mostly species-dependent (Table 4). A single strain of *M. pulcherrima* was the most tolerant among 36 strains used in this study. Similar findings regarding *M. pulcherrima* tolerance to hydrogen peroxide are reported by Grazia et al. (2019) and Mestre Furlani et al. (2017). *P. anomala* and *Kloeckera* strains also exhibited high tolerance to peroxide stress in this study, which is in agreement with the data reported by Mestre Furlani et al. (2017).

**Table 4.** Tolerance of non-conventional yeast strains to hydrogen peroxide

Yeast species	Strain number UCM Y-	Diameter zone, mm*		
		Hydrogen peroxide concentration		
		0.25 M	0.5 M	1.0 M
*Torulaspora* *delbrueckii*	2737	33.67 ± 1.15^fgij^	43.67 ± 1.15 ^gj^	49.0 ± 1.0^fijp^
	2738	36.67 ± 1.15^efhi^	49.33 ± 0.58^def^	57.33 ± 0.58^de^
	2739	28.67 ± 0.58^bfgimn^	34.33 ± 1.15^cip^	51.33 ± 1.52^efip^
	2741	40.0 ± 1.73^deh^	44.33 ± 0.58^dgjk^	51,67 ± 1.52^efijlp^
	2749	19.67 ± 1.15^bklmo^	22.33 ± 1.15^lnosu^	29.67 ± 2.08^cklmr^
*Kloeckera apiculata*	2728	14.33 ± 0.58^lo^	16.33 ± 0.58 ^ms^	27.67 ± 0.58^klmrt^
	2729	15.67 ± 1.15^klo^	17.67 ± 0.58 ^ms^	27.0 ± 1.0^klmqrt^
*K. javanica*	2689	16.67 ± 0.58^cklo^	18.33 ± 0.58 ^ms^	24.33 ± 0.58^lqrut^
	2693	14.33 ± 0.58^lo^	18.0 ± 2.0 ^ms^	22.0 ± 1.73^tu^
	2695	15.0 ± 1.0^klo^	18.33 ± 0.58 ^ms^	26.0 ± 1.0^klqrt^
*Candida inconspicua*	2732	42.0 ± 2.0^de^	56.0 ± 1.0^afhr^	67.33 ± 1.52 ^g^
*C. lambica*	2696	18.33 ± 0.58^klmo^	23.0 ± 1.73 ^l,nosu^	27.33 ± 1.15^klmqrt^
*C. valida*	969	22.0 ± 1.73^bckmno^	28.0 ± 1.73^cn^	33.33 ± 0.58^bckm^
	971	26.33 ± 0.58^bcgjmn^	26.0 ± 1.0^bnou^	30.0 ± 1.0^cklmr^
	973	24.0 ± 1.0^bcjkmn^	28.67 ± 0.58^bcnp^	36.33 ± 0.58^bco^
*C. vini*	997	15.33 ± 1.15^klo^	20.67 ± 0.58^lmosu^	27.0 ± 1.73^klmqrt^
	998	29.67 ± 1.53^fgijn^	33.67 ± 0.58^cip^	45.0 ± 1.0^hnp^
*Metschnikowia pulcherrima*	333	0^p^	0 ^t^	13.67 ± 1.15^s^
*Pichia membranifaciens*	471	51.67 ± 1.53^a^	59.0 ± 2.0^ar^	81.33 ± 1.53^a^
	473	41.0 ± 2.0^deh^	52.67 ± 0.57^efhr^	54.33 ± 0.57^defj^
	477	41.67 ± 1.53^deh^	57.67 ± 1.53^ahr^	60.0 ± 1.0^d^
	480	42.33 ± 1.15^de^	47.67 ± 0.57^dek^	57.0 ± 1.0^d^
	481	37.33 ± 1.53^dehi^	49.67 ± 2.08^def^	58.67 ± 0.57^d^
	482	32.0 ± 2.0^fghij^	42.33 ± 1.15^gjkq^	52.0 ± 1.0^efij^
	2733	30.67 ± 1.53^fgijn^	39.67 ± 1.53^gq^	48.0 ± 1.0^hijp^
	2734	52.67 ± 1.53^a^	55.0 ± 1.73^fhr^	68.33 ± 0.58 ^g^
	2735	49.33 ± 0.58^a^	53.0 ± 1.73^efhr^	69.33 ± 1.15^g^
*P. anomala*	212	15.67 ± 0.58^klo^	19.67 ± 1.53^lmosu^	23.67 ± 1.15^lqt^
	213	17.67 ± 0.58^cklmo^	19.33 ± 0.58^lmos^	27.33 ± 0.58^klmqrt^
	215	17.67 ± 1.15^klmo^	21.67 ± 1.53^lmosu^	27.67 ± 0.58^klmrt^
	216	15.33 ± 0.58^klo^	20.0 ± 1.73^lmos^	30.33 ± 1.53^cklmr^
	217	22.33 ± 1.53^bkmo^	22.33 ± 0.57^lns^	27.66 ± 1.15^klmrt^
*Zygosaccharomyces bailii*	657	27.66 ± 0.58^bfgjn^	32.0 ± 1.73^bcip^	41.33 ± 1.53^hno^
*Z. bisporus*	2730	21.33 ± 1.53^bckmno^	30.66 ± 0.58^bcip^	33.33 ± 1.53^bckm^
*Z. bisporus*	2731	24.0 ± 1.0^bckmn^	29.0 ± 1.0^bcnp^	35.0 ± 1.0^bco^
*Z. fermentati*	658	30.0 ± 1.0^fgijn^	28.0 ± 1.0^bcn^	38.67 ± 1.53^bno^

*Means within the same column with the same letter are not significantly different at p ≤ 0.05

All *P. anomala* and *T. delbrueckii* strains and a single strain of *M. pulcherrima* were tolerant to osmotic stress caused by 50% glucose (Table 5). This is consistent with the findings in the study by Mukherjee et al. (2017) that indicated the high osmotic tolerance in these species. Seventeen out of 36 yeast strains (*P. membranifaciens* and several strains of *Candida* and *Zygosaccharomyces*) used in the current study lacked the ability to grow at 50% glucose, which is similar to the results obtained by Grazia et al. (2019). These authors reported that 13 out of 29 non-*Saccharomyces* wine yeasts could not grow in the medium containing 40% glucose.

**Table 5.** Enological traits of non-conventional yeast strains

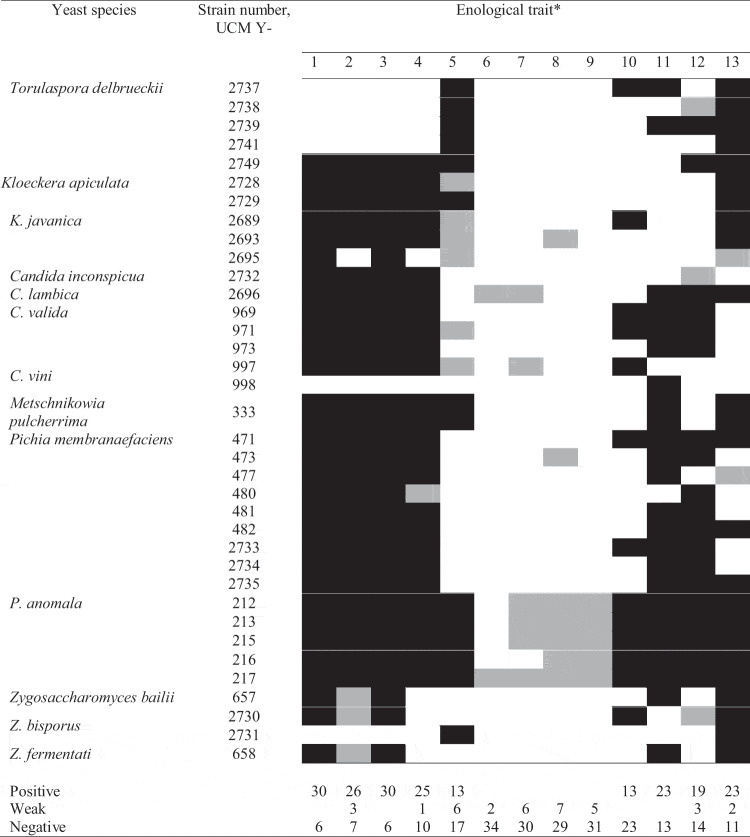

* Tolerance to stress factors: 1–200 μM CuSO_4_ (copper sulfate), pH 6.0, 2–200 μM CuSO_4_, pH 3.5, 3–400 μM CuSO_4_, pH 6.0, 4–400 μM CuSO_4_, pH 3.5; osmotolerance: 5–50% glucose; biogenic amine production: 6–1% amino acid mix, 7–2% amino acid mix; killer activity: 8 – against *Saccharomyces cerevisiae* UCM Y-554, 9 – against *Kluyveromyces marxianus* UCM Y-1591; acid production from glucose: 10 – chalk solubilisation on Causter’s agar; organic acid assimilation: 11 – malic acid, 12 – acetic acid; fermentative activity: 13 – CO_2_ production in semi-synthetic fermentation medium. Black colour indicates positive reaction or tolerance, grey colour indicates weak reaction or tolerance, white colour indicates negative reaction or sensitivity

Copper-containing chemicals are widely used as traditional fungicides in agriculture and can be toxic to yeasts resulting in the inhibition of growth and activity during alcoholic fermentation (Capece et al. 2018). Consequently, tolerance to copper would be a valuable characteristic for yeasts used in fermentation. Most yeast strains used in this study were tolerant to copper sulfate at 200 and 400 µM at pH 6.0 and 3.5, while *T. delbrueckii, C. vini* and *Z. bisporus* strains lacked tolerance to copper (Table 5). Grazia et al. (2019) reported lower levels of copper tolerance in non-conventional yeasts of enological origin, none of the isolates tolerated CuSO_4_ above 300 µM. However, most *M. pulcherrima* strains isolated from wineries tolerated up to 2 mM copper (Barbosa et al. 2018) and high resistance to copper up to 10 mM was reported for *Starmerella bacillaris* and *Metschnikowia* yeasts isolated from high-sugar habitats (Binati et al. 2019). The low copper tolerance of *T. delbrueckii* strains demonstrated in this study is in accordance with data reported by Gava et al. (2016).

Killer activity in wine yeasts could be a beneficial trait helping starter cultures to inhibit the undesirable fungal microbiota in must (Zagorc et al. 2001). Several studies have focused on the killer potential of non-*Saccharomyces* yeasts as a way to prevent unwanted yeast growth (Yap et al. 2000; Comitini et al. 2011). However, none among 36 yeast strains exhibited a pronounced killer activity against two killer-sensitive strains *S. cerevisiae* and *K. marxianus* (Table 5). Weak killer activity was detected in several yeasts, mostly *P. anomala* strains. *Pichia/Wickerhamomyces anomalus* strains of enological origin were reported to possess killer activity in several studies (Yap et al. 2000; Zagorc et al. 2001; Sangorrín et al. 2008).

Wine yeasts should lack the ability to produce high quantities of organic acids, including acetic, low levels of volatile organic acids are especially important during the production of botrytized wines (Magyar and Tóth 2011). Thirteen out of 36 yeast strains produced a visible zone of solubilisation on carbonate agar, which included all *P. anomala* strains, 2 strains of *C. valida* and *P. membranifaciens*, 1 strain of *T. delbrueckii, K. javanica, C. vini*, and *Z. bisporus* (Table 5). Low incidence of organic acid production by wine yeasts was also reported in other studies (Suárez Valles et al. 2008; Di Maio et al. 2012). However, the production of organic acids by several yeast strains in this study could be, in turn, mitigated by the ability of these strains to utilize organic acids (malic and acetic). Most *P. membranifaciens* strains and all *C. valida* and *P. anomala* strains could assimilate malic and acetic acids as a sole source of carbon.

Some non-*Saccharomyces* wine yeasts are known to produce undesirable compounds such as biogenic amines that are at high levels toxic for humans (Ivit and Kemp 2018). Weak ability to produce biogenic amines from a mixture of seven amino acids was detected in six out of 36 strains – *C. lambica, C. vini* and *P. anomala* (Table 5). Non-conventional wine isolates as well as wine yeasts *S. cerevisiae* are capable of producing biogenic amines during wine production (Guo et al. 2015). The quantitative determination of main biogenic amines (histamine, tyramine, putrescine, cadaverine and phenylethylamine) generated during fermentation by the selected yeast strain or strains would be required in order to guarantee the safety of the final product.

The ability of non-conventional yeasts to carry out fermentation at low pH was mostly species-dependent (Table 5), as all *T. delbrueckii, Zygosaccharomyces* and *P. anomala* strains displayed strong fermentative activity in the preliminary screening, while all *C. valida* and *C. vini* strains were non-fermenting. Many non-conventional yeasts are weakly fermentative or lack the ability to ferment sugars altogether (Padilla et al. 2016). Similarly, comparatively high fermentation power was detected in *T. delbrueckii* strains by Comitini et al. (2011) and Vigentini et al. (2016), and in *Zygosaccharomyces* yeasts by Domizio et al. (2011), while weak fermentative power was demonstrated by *Candida* yeasts (Comitini et al. 2011). Interestingly, in the preliminary screening, we observed high fermentative activity in *P. anomala* strains, although *Pichia* yeasts are usually regarded as weakly fermentative (Padilla et al. 2018).

**Figure 2.**
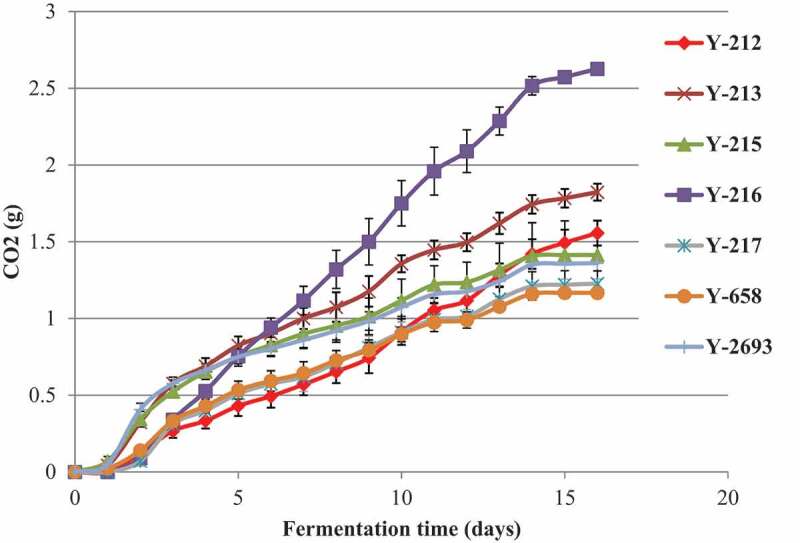
CO_2_ production by non-conventional yeasts in semi-synthetic medium. Results are presented as means of triplicates with the corresponding standard deviation (± SD)

Seven non-conventional yeast strains *P. anomala* Y-212, Y-213, Y-215, Y-216, Y-217, *Z. fermentati* Y-658 and *K. javanica* Y-2693 were further characterised in microfermentation experiments as they possessed tolerance to ethanol, osmotic and peroxide stress, extracellular hydrolases of enological value (β-glucosidase, protease, esterase, xylanase) and high fermentative activity in the preliminary screenings. Strain *M. pulcherrima* was excluded from microfermentation trials due its poor ethanol tolerance at low pH, while *T. delbrueckii* and *Zygosaccharomyces* strains mostly did not display extracellular hydrolases of enological value. All tested strains produced comparatively low levels of CO_2_ (1.17–2.63 g per 100 ml after 16 days of fermentation) (Figure 2). The highest fermentative activity in semi-synthetic media was demonstrated by *P. anomala* Y-216 (2.63 ± 0.01 g of CO_2_, p < 0.05) that corresponds to 3.29% (v/v) ethanol in the medium (Vaughan-Martini and Martini 1998).

As the obtained data indicate that the selected yeast strains lack the ability to complete fermentation on their own, the use of these yeasts in co-culture with *S. cerevisiae* strain in the mixed wine fermentation would be advisable. As yeast strain *P. anomala* Y-216 displayed high tolerance to ethanol, copper sulfate, osmotic and peroxide stress, strong lipase, esterase, xylanase, and β-glucosidase activities and also the highest fermentative activity among the tested yeasts, it was selected in this preliminary screening work as a potential co-culture to use alongside *S. cerevisiae* in wine fermentation.

## Conclusion

*S. cerevisiae* yeasts are the principal performers of alcoholic fermentation during wine production, but are not usually considered as strong producers of many extracellular hydrolases, including proteases, lipases, esterases, cellulases and some others. This study revealed that non-conventional yeasts of enological origin could potentially be exploited as producers of several extracellular hydrolases. *P. anomala* strains were especially promising in this aspect exhibiting strong lipase, esterase, xylanase and β-glucosidase activities and *M. pulcherrima* as a producer of lipase, protease and β-glucosidase. Several *P. anomala* strains demonstrated valuable enological traits, such as a wide range of enzymatic activities, high stress tolerance, killer activity, and utilization of organic acids. However, due to their limited fermentative efficiency, the use of mixed cultures with highly fermentative *S. cerevisiae* yeasts would be more preferable. In conclusion, this study represents the first step for selecting a non-conventional yeast strain of enological origin as a potential co-culture for winemaking.
